# Neural correlates of uncertain decision making: ERP evidence from the Iowa Gambling Task

**DOI:** 10.3389/fnhum.2013.00776

**Published:** 2013-11-15

**Authors:** Ji-fang Cui, Ying-he Chen, Ya Wang, David H. K. Shum, Raymond C. K. Chan

**Affiliations:** ^1^Information center, National Institute of Education SciencesBeijing, China; ^2^Institute of Developmental Psychology, School of Psychology, Beijing Normal UniversityBeijing, China; ^3^Neuropsychology and Applied Cognitive Neuroscience Laboratory, Key Laboratory of Mental Health, Institute of Psychology, Chinese Academy of SciencesBeijing, China; ^4^Behavioural Basis of Health Program, Griffith Health Institute and School of Psychology, Griffith UniversityBrisbane, QLD, Australia

**Keywords:** uncertain decision making, Iowa Gambling Task, emotion, ERP, somatic marker hypothesis

## Abstract

In our daily life, it is very common to make decisions in uncertain situations. The Iowa Gambling Task (IGT) has been widely used in laboratory studies because of its good simulation of uncertainty in real life activities. The present study aimed to examine the neural correlates of uncertain decision making with the IGT. Twenty-six university students completed this study. An adapted IGT was administered to them, and the EEG data were recorded. The adapted IGT we used allowed us to analyze the choice evaluation, response selection, and feedback evaluation stages of uncertain decision making within the same paradigm. In the choice evaluation stage, the advantageous decks evoked larger P3 amplitude in the left hemisphere, while the disadvantageous decks evoked larger P3 in the right hemisphere. In the response selection stage, the response of “pass” (the card was not turned over; the participants neither won nor lost money) evoked larger negativity preceding the response compared to that of “play” (the card was turned over; the participant either won or lost money). In the feedback evaluation stage, feedback-related negativity (FRN) was only sensitive to the valence (win/loss) but not the magnitude (large/small) of the outcome, and P3 was sensitive to both the valence and the magnitude of the outcome. These results were consistent with the notion that a positive somatic state was represented in the left hemisphere and a negative somatic state was represented in the right hemisphere. There were also anticipatory ERP effects that guided the participants' responses and provided evidence for the somatic marker hypothesis with more precise timing.

## Introduction

Uncertainty can be defined as an imperfect knowledge about the outcome that will follow a choice (Platt and Huettel, 2008). Uncertainty exists in some form in all daily life choices, i.e., we are always engaging in uncertain decision making. For example, if you decide to step into an unfamiliar restaurant for dinner, you may receive delicious food and feel happiness, or you may be disappointed and feel regret.

In the laboratory, the Iowa Gambling Task (IGT) (Bechara et al., 1994) simulates the uncertainty of decision making of gains and losses in real life and provides a useful tool to examine uncertain decision making (Toplak et al., 2010). In the IGT, a participant is presented with four decks of cards, and each card contains a reward and a punishment. The participant is asked to pick cards from these decks to earn as much money as possible. Decks A and B contain a large win each time and sometimes an even larger loss; in the long term, the participant would lose money if these decks continued to be selected (the disadvantageous decks). Decks C and D contain a small win each time and sometimes a small loss; in the long term, the participant would earn money if these decks continued to be selected (the advantageous decks)^1^.

Healthy participants will learn which decks are advantageous and will select more often from these decks, while patients with ventromedial prefrontal (VMPFC) lesions will persist in selecting from the disadvantageous decks that provide a large immediate reward (Bechara et al., 1996, 1997). More interestingly, healthy comparisons showed anticipatory skin conductance responses (SCRs) when they choose decks, and the SCRs were higher when choosing disadvantageous decks; however, the VMPFC patients did not show the same anticipatory SCRs (Bechara et al., 1996, 1997). After ~80 trials, most healthy participants knew which decks were good and which decks were bad. Some patients also reached this explicit knowledge about the task, but they still tended to choose the disadvantageous decks without showing anticipatory SCRs (Bechara et al., 1997). These results indicated that although VMPFC patients understood the task and had some explicit knowledge about the decks, they could not generate an automatic emotional response to guide them to a better decision (Bechara et al., 1997).

Based on their studies in VMPFC patients (e.g., Damasio et al., 1991; Damasio, 1994) proposed the famous somatic marker hypothesis; he argued that these patients had decision-making deficits because they were not able to use somatic markers to guide their decision making. The somatic markers are body-generated, emotion-based signals (see also Dunn et al., 2006). According to the hypothesis, specific internal and external stimuli will cause changes in the body and brain (peripheral and central representations); these changes converge to and compose the emotion. The somatic signals projected to the brain represent the basic element that will be perceived as a feeling. These emotions and feelings are associated by learning and can be used to predict future outcomes in specific situations. The SCR studies (Bechara et al., 1996, 1997) on the IGT provided key evidence for the somatic marker hypothesis (Dunn et al., 2006). Given that SCRs represent physiological indicators of changes of somatic states, the fact that the patients did not show anticipatory SCRs suggests that when facing an imagined scenario or situation, the patients could not change their somatic states. Thus, the fact that they could not enact a somatic state proper to the outcome of a response is the reason that they did not choose advantageously, i.e., myopia for the future (Bechara et al., 2000).

However, there are several limitations of the SCR studies. First, in the psychophysiology analysis, the deck that participants selected at last was used to designate each anticipatory “somatic marker,” however, in the deck selection phase, participants were free to shift their attention across all decks prior to selecting one. This procedure meant that the anticipatory SCRs may not reflect attention to a single card but shifting attention across all decks before making a choice (Dunn et al., 2006). Second, a study using the IGT in rhesus monkeys showed that SCRs were associated with the anticipation of a reward after a decision had been made rather than reflecting the decision-making process directly (Amiez et al., 2003). Thus, due to the low temporal resolution of SCRs, it was difficult to separate the signal related to response selection from the anticipation of feedback after the response (Dunn et al., 2006). One solution is to use other psychophysiological responses with a faster time course, such as event-related potentials (ERPs).

Many ERP studies have been performed on decision making (Bland and Schaefer, 2011). Decision making can be divided into three stages from an information-processing perspective: I, choice evaluation; II, response selection; III, feedback processing (Fang et al., 2009). However, most ERP studies on the IGT have focused on the third stage, i.e., the modulations related to feedback. There were mainly two modulations related to feedback: feedback-related negativity (FRN) and P3.

Gehring and Willoughby (2002) found that negativity was present ~265 ms following the feedback of a win or a loss and that the source was located in the medial frontal cortex. They named this phenomenon MFN (medial frontal negativity), and later studies called it FRN. The FRN was sensitive to loss; the magnitude was larger for a loss than a win. Subsequent studies revealed that FRN and P3 were differentially modulated by the valence and the magnitude of the outcome. FRN was sensitive to the valence but not the magnitude of the feedback, i.e., a loss would cause a larger amplitude than a win, but the magnitude of a loss or win did not have an effect on the amplitude of the FRN (Gehring and Willoughby, 2002; Yeung and Sanfey, 2004; Toyomaki and Murohashi, 2005; Holroyd et al., 2006; Wu and Zhou, 2009). These results indicated that FRN reflected the early appraisal of feedback on a binary classification basis of good vs. bad outcomes. Studies found that P3, which showed the largest amplitude at central and parietal regions, was sensitive to both magnitude and valence (Toyomaki and Murohashi, 2005; Hajcak et al., 2006; Hewig et al., 2007). P3 may reflect a later, top-down controlled feedback evaluation process, in which factors related to the allocation of attentional resources played a role.

Only two ERP studies have examined the response selection stage (ERP modulations before the response was made) of decision making using the IGT. Carlson and Zayas (2009) conducted an ERP study using a children's version of the IGT, the Hungry Donkey Task (HDT), in 8-year-old children. In this study, participants were asked to select one out of four doors for a hungry donkey; behind each door, there were wins and losses of apples. The participants were asked to earn as many apples as possible for the donkey. The results showed anticipation effects preceding door choice, i.e., ERP waveforms 300–150 ms prior to response selection were more negative for disadvantageous door selections. These results indicated that children showed an anticipation effect for the doors that were associated with losses and were disadvantageous. Another study used the IGT in adults and named the negativity preceding movement (response) the Decision Preceding Negativity (DPN) (Bianchin and Angrilli, 2011). The authors found that the DPN was more negative over the right frontal regions when participants selected the disadvantageous decks than the advantageous decks. These results suggested a role for emotion anticipation in making decisions.

One limitation of these two ERP studies is that the response selection stage may have been confounded with choice evaluation because in their version (free choice version) of the IGT, participants were presented with four decks of cards and asked to select one card at a time. Thus, the choice evaluation and the response stage cannot be separated. To overcome this shortcoming, we adopted a modified version of the IGT [single choice version, adapted from Cauffman et al. (2010)] in the present study; participants were presented with one card from a deck at a time and asked to decide whether to play or pass that card. After a response was made, the outcome of the current trial would be presented. Using this paradigm, we could evaluate all three stages (choice evaluation, response selection, and feedback evaluation) of uncertain decision making.

For the present study, we had the c, based on Bechara et al.'s (1996) assumption, in normal individuals, the brain contains neural circuitry that links the stimulus configuration of a given deck to the representations of goodness and badness. On the other hand, the right frontal hemisphere is sensitive to punishment learning and negative situations, whereas the left frontal hemisphere is sensitive to reward learning and positive situations (Cunningham et al., 2005; Dunn et al., 2006; Ohgami et al., 2006). Positive and negative somatic states are represented in the frontal areas of the left and right hemisphere, respectively (Bechara and Damasio, 2005; Dunn et al., 2006). Thus, we predicted that in the choice evaluation stage, the advantageous decks would evoke more activity in the left frontal hemisphere, and the disadvantageous decks would evoke more activity in the right frontal hemisphere, given that the decks are associated with positive and negative somatic (emotional) representations.

Second, regarding the anticipation effect in the response selection stage of decision making, two studies (Carlson and Zayas, 2009; Bianchin and Angrilli, 2011) found that the amplitudes of the readiness potential or the DPN over the right frontal regions evoked by the disadvantageous decks were larger than those of the advantageous decks, indicating that risky decisions would evoke a stronger anticipation effect. We adopted a single choice version of the IGT and asked the participants to decide to “play” or “pass” a card from a deck. We predicted that, consistent with the previous studies, anticipation would also be associated with responses; specifically, the response of “pass” would evoke larger right frontal DPN than “play” because of the stronger somatic states. If the participants perceived the trial to be risky and accompanied by a greater physiological arousal, they would tend to “pass”; if the participants perceived the trial to be safe, they would tend to “play.” Thus, the response “pass” would show larger DPN than “play.” Furthermore, for disadvantageous decks, if the participants perceived the trial to be safe and a smaller physiological response occurred, they would choose to “play” (there were many win trials in disadvantageous decks) and show a small DPN.

Third, for the feedback evaluation stage, we predicted that the results would be consistent with previous studies in that the FRN would only be sensitive to the valence of the outcome, whereas the P3 would be sensitive to both the valence and the magnitude of the outcome.

## Methods

### Participants

Twenty-eight university students participated in this study; two participants were excluded from the analysis because of data recording problems. The remaining 26 participants consisted of 12 females and 14 males. Their age ranged from 19 to 25 years with a mean age of 22.35 years (*SD* = 1.74). None of the participants had a history of mental illness, neurological illness, brain injury, or drug/alcohol dependence. All participants had normal or corrected to normal vision. Two participants were left handed, whereas the rest were right handed measured by a handedness questionnaire (Annett, 1970). An initial analysis showed that handedness did not affect the results; thus, handedness was not considered in further analysis. The present study was approved by the ethical committee of the School of Psychology, Beijing Normal University. Participants provided written informed consent before the study.

### Experimental task

The modified IGT used in this study was adapted from Cauffman et al. (2010). In this task, the participants need to make a play/pass decision on one of the four decks preselected by the computer on each trial.

To reduce eye movement during the experiment, the four decks were presented on the screen in two rows. The decks were labeled A, B, C, and D, but these labels were only for participants to discriminate the cards and were unrelated to advantageous or disadvantageous decks. The advantageous and disadvantageous decks were randomly assigned to these labels. When we refer to deck A, B, C, or D later in this paper, we mean that decks A and B were disadvantageous decks and that decks C and D were advantageous decks, as in the original version of the IGT.

**Figure 1:**
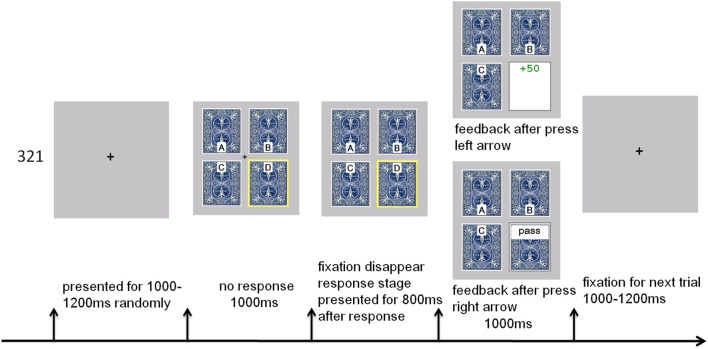
**Experimental flow of the IGT task**.

The experimental flow is presented in Figure 1. Each trial included three processing stages. In the stimulus presentation (choice evaluation) stage, a fixation in the middle of the four decks was presented for 1000 ms. One of the four decks was highlighted with a yellow border, which meant that one card was selected from that deck. Participants were required to decide to play or pass that card, however, they could not make responses during this period. In the response selection (decision execution) stage, the fixation disappeared, and participants were required to make a response. The participants could press the “←” key if they wanted to play or the “→” key if they wanted to pass. Feedback (feedback evaluation stage) for the current trial was presented 800 ms after the response. The feedback was presented on the screen for 1000 ms, followed by a fixation presented for 1000–1200 ms, and then the next trial appeared. The participants began with a loan of 2000 Yuan. The running total of the participant's “earnings” was presented at the end of each block (20 trials). Each of the four decks was randomly preselected five times for participants to make a decision in each block. To reduce eye movement and head movement, the feedback was presented near the center of the screen (see Figure 1). We used the same outcome feedback format as Cauffman et al. (2010), i.e., participants received the net gain or loss of a card, rather than presenting both a gain and a loss. If the outcome was “win,” the feedback was presented with a green positive value (e.g., +50); if the outcome was “loss,” the feedback was presented with a red negative value (e.g., −100). If the participant chose to pass, “pass” was presented on the screen as feedback. A previous study showed that the color of the feedback did not affect the EEG data (Martín et al., 2010). To obtain enough trials for averaging in each condition, we set the total number of trials to 400, divided into 20 blocks. Each block included a 3 s countdown to remind the participants to prepare themselves.

It was emphasized to participants before the experiment that each deck had its own rules and had enough cards to ensure that it would not be exhausted during the trial. The cards in each deck were presented in a fixed pseudorandom order; the “passed” cards remained at the top of that deck and would be presented the next time if the participant chose to play with that deck (participants were not informed of this procedure). This setting was to ensure that the expected value of each deck was the same as designed and the same for each participant. The payoff schedules for each deck reflected the net outcomes and were changed slightly from the original IGT^2^. Two of the decks (C and D) were advantageous and produced a net gain over repeated play. The expected values for 10 consecutive cards of decks C and D were 200 and 250, respectively^3^. The other two decks were disadvantageous (A and B) and caused a net loss over repeated play. The expected values for 10 consecutive cards of decks A and B were both −250. In addition, within each type of deck (advantageous and disadvantageous), one deck (B or D) presented an infrequent (10%) but relatively large loss, whereas the other deck (A or C) presented a frequent (50%) but relatively small loss.

### Procedure

The participants were first introduced to the experiment and given an opportunity to ask questions. After an informed consent was signed, the study began. The distance from the participant's eyes to the screen was 60 cm; the visual angle was ~4.7°. Participants were told that they would be paid based on their performance before the formal study began. Participants were allowed a rest between every 2 blocks. The EEG recording lasted 50–60 min. The whole experiment lasted 1.5–2 h, including preparation and debriefing.

### EEG recording and analysis

The EEG data were recorded with a band pass of 0.05–100 Hz and a sampling rate of 500 Hz from 64 scalp sites using Ag/AgCl electrodes (NeuroScan Inc., Herndon, Virginia, USA). The left mastoid was used as a reference in recording and was transformed to the average of the left and right mastoids reference offline. Horizontal electrooculogram (EOG) recording electrodes were positioned at the outer canthi of the left and right eyes; vertical EOG recording electrodes were positioned above and below the left eye. The impedances of all electrodes were kept below 5 kΩ through the experiment.

After the eye movement reduction (ocular reduction in the NeuroScan software 4.3 version), a 30 Hz zero-phase-shift low-pass filter, 24 dB/oct, was used for the offline digital filter with the EEG data. Trials with artifacts larger than ±100 μV were rejected.

EEG data were analyzed based on the processing stages of decision making:

Choice evaluation stage: the epoch was set to 200 ms prior to the stimulus onset and 1000 ms after the stimulus onset, with the baseline from 200 ms prior to stimulus onset to stimulus onset. The main modulation we analyzed was P3 (300–500 ms). We conducted three analyses on P3 to examine whether the disadvantageous and advantageous decks would evoke different patterns of P3 in the left and the right hemisphere at this stage: (1) The global difference in P3 between the advantageous and disadvantageous decks; (2) The change in ERP modulation differences between the advantageous and disadvantageous decks over the course of the task; (3) ERP modulations in the choice evaluation stage with subsequent different responses (play, pass). Nine electrodes (F5, FZ, F6, C5, CZ, C6, P5, PZ, P6) were used for analysis.

Response selection stage: as in Carlson and Zayas (2009), the preliminary analysis used the average amplitude during −1000 to 800 ms as the baseline; the major fluctuations in ERP amplitudes occurred before the response (DPN) and were relatively stable after the response (particularly 200 ms before feedback, i.e., 600–800 ms after the response). Thus, in the formal analysis, we used the 600-800 ms after the response as the baseline and the 800 ms preceding the response as the analysis time window for DPN (we also referred to the average response time of 782 ms to set the time window). We analyzed whether different responses (“play” or “pass”) would show different DPN. The same nine electrodes were used for this analysis as in the previous stage.

Feedback evaluation stage: we focused our analyses on the effects of the valence and the magnitude of feedback on the FRN and P3. According to the valence and the magnitude of the outcome, the feedback was divided into four categories: large loss (losses of 100 or more; appeared in decks A, B, and D), small loss (losses of less than 100; appeared in decks A, and C), large win (wins of 90 or more; appeared in decks A and B), small win (wins of less than 90; appeared in decks C and D). Based on our results and previous studies, the mean amplitude during 300–450 ms was selected to capture P3. Consistent with previous studies, the amplitude of the FRN was affected by P2; as in Toyomaki and Murohashi (2005), we adopted the difference between the positive peak of 150–200 ms and the negative peak of 220–330 ms as the amplitude of the FRN. From our results and consistent with previous studies (Li et al., 2010; Rigoni et al., 2010), the prominent difference lies in the midline electrodes. Thus, we analyzed the FZ, FCZ, and CZ for FRN, and the CZ, CPZ, and PZ for P3. The analysis on amplitude was performed using SPSS 18.0; all analyses used the Greenhouse-Geisser correction.

### Behavioral data analysis

For behavioral results, we conducted two main analyses: first, the net score (the number of plays with advantageous decks minus the number of plays with disadvantageous decks) over the course of the task; second, the profile of the response for each deck of cards.

## Results

### Behavioral results

#### Net score in each block

**Figure 2:**
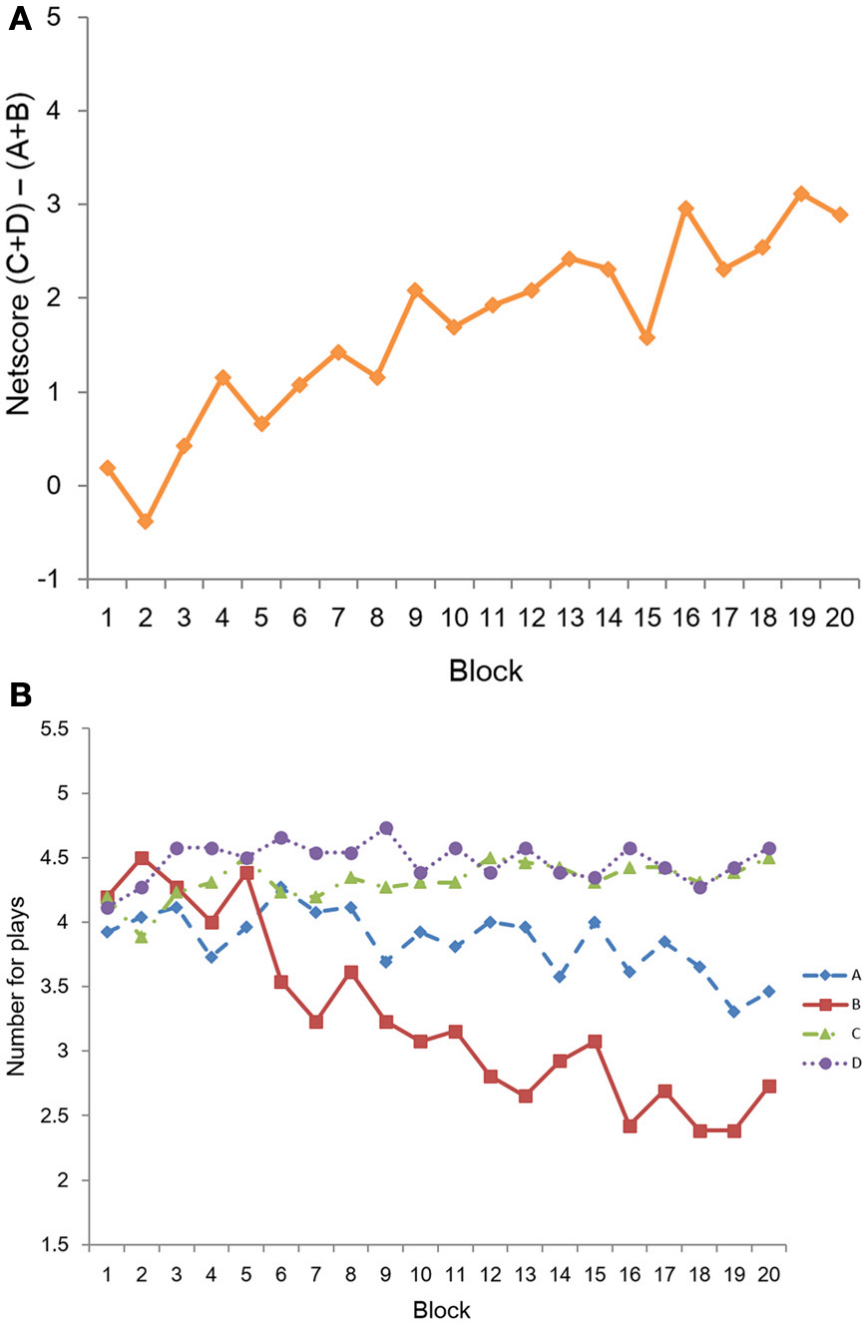
**Behavioral performance in the IGT. (A)** Net score in each block. **(B)** The number of plays in each block for each deck.

To explore the trend of net score over the course of the task, a repeated ANOVA revealed that the Block effect was significant, *F*_(19, 475)_ = 5.89, *p* < 0.001, η^2^_*p*_ = 0.191 (see Figure 2A). From the figure, we can see that over the course of time, the proportion of “play” responses with the advantageous deck increased. Further analysis showed that the block net score was significantly larger than 0 from block 6 to 20 (*t*-value ranged from 2.9 to 5.6; to adjust for multiple comparisons, we set *p* < 0.01).

#### Number of plays in the IGT

The number of plays in each deck is presented in Figure 2B. The proportion of play for each deck was 89.4% (D), 86.5% (C), 77.1% (A), and 65.2% (B). A 4 (Deck) × 20 (Block) repeated ANOVA revealed that there was a significant main effect of Deck, *F*_(3, 75)_ = 18.38, *p* < 0.001, η^2^_*p*_ = 0.424. *Post-hoc* analysis revealed that the number of plays in decks D and C were significantly larger than decks A and B, and deck A was more often chosen to play than deck B. There was also a significant main effect of Block, *F*_(19, 475)_ = 3.64, *p* < 0.001, η^2^_*p*_ = 0.127. The Deck × Block interaction was significant, *F*_(57, 1425)_ = 3.68, *p* < 0.001, η^2^_*p*_ = 0.128. From Figure 2B, we saw different trends for each deck: the number of plays with deck D remained high throughout the task, while the number of plays for deck C showed a trend toward an increase over the course. For deck A, the number of plays showed a trend toward a decrease, while deck B had the most significant trend of decreasing the number of plays. These results indicated that participants showed a learning effect in the task.

### ERP results

#### Choice evaluation stage

(1) The global difference in the ERP modulations between advantageous and disadvantageous decks

**Figure 3:**
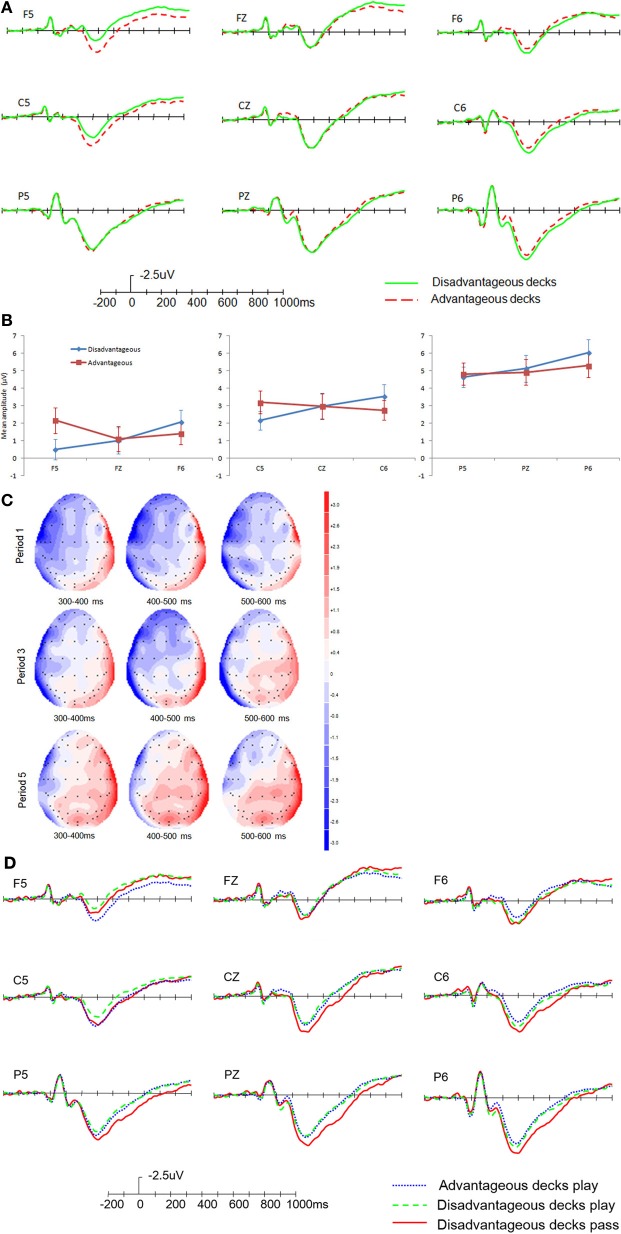
**ERP results in the choice evaluation stage. (A)** Global difference between advantageous and disadvantageous decks. **(B)** Amplitudes of disadvantageous decks and advantageous decks in different regions. **(C)** Topographical maps for the difference wave between disadvantageous decks and advantageous decks in different periods of the task. **(D)** Comparison of modulations between different responses. Error bars indicate the standard error.

The grand averageof advantageous and disadvantageous decks in the choice evaluation stage was presented in Figure 3A.

We analyzed the mean amplitude of P3 on 9 electrodes (F5, FZ, F6, C5, CZ, C6, P5, PZ, P6). A 2 (Type of deck: advantageous, disadvantageous) × 3 (Region: frontal, central, parietal) × 3 (Laterality: left, middle, right) repeated ANOVA was conducted. Only the results of the main effect of Type of deck and the interactions involving Type of deck were presented here. The mean numbers of artifact-free trials for analysis were 197 and 196 for disadvantageous and advantageous decks, respectively.

There was a significant interaction between Type of deck and Region, *F*_(2, 50)_ = 8.29, *p* = 0.001, η^2^_*p*_ = 0.249; Type of deck and Laterality, *F*_(2, 50)_ = 7.20, *p* = 0.002, η^2^_*p*_ = 0.224; the three-way interaction of Type of deck × Region × Laterality was also significant, *F*_(4, 100)_ = 5.79, *p* = 0.003, η^2^_*p*_ = 0.188. Further analysis of the three way interaction revealed that the interaction between Type of deck and Laterality showed significant differences in different regions, i.e., the interaction was significant in the frontal (*p* = 0.007) and central (*p* = 0.009) regions and marginally significant in the parietal region (*p* = 0.060). The significant interactions revealed a larger P3 for advantageous decks in the left hemisphere and a larger P3 for disadvantageous decks in the right hemisphere (see Figure 3B). The results also showed that the P3 amplitude difference between advantageous and disadvantageous decks in the left hemisphere decreased from the frontal to the parietal region, with a significant difference in the frontal (*p* = 0.005) and central (*p* = 0.027) regions but not in the parietal region (*p* = 0.580). In the right hemisphere, the differences between the advantageous and disadvantageous decks were significant in the central (*p* = 0.027) and the parietal regions (*p* = 0.029) and marginally significant in the frontal region (*p* = 0.095). The interaction between Type of deck and Laterality was consistent with the emotional asymmetry hypothesis that positive emotion evoked more activity in the left brain, and negative emotion evoked more activity in the right brain (Cunningham et al., 2005; Dunn et al., 2006; Ohgami et al., 2006).

(2) Change of ERP differences between the advantageous and disadvantageous decks in different periods of the task

To explore whether the ERP modulations changed over the course of the task, we divided the task into five periods; each period consisted of four blocks (80 trials). See Figure 3C for topographical maps of periods 1, 3, and 5.

A 5 (Period of task: period 1, period 2, period 3, period 4, period 5) × 3 (Region: frontal, central, parietal) × 3 (Laterality: left, middle, right) repeated ANOVA was conducted on the difference amplitude of P3 (the advantageous decks subtracted from the disadvantageous decks). The results showed that the main effect of Period of task (*p* = 0.142) and the interactions involving this variable (*p* ranged from 0.323 to 0.751) were non-significant. However, as shown in Figure 3C, the difference amplitude decreased in the left hemisphere and increased in the right hemisphere over the course of the task.

(3) ERP modulations in the choice evaluation stage with different subsequent responses

The best choice for the disadvantageous decks A and B was to “pass.” However, the behavioral responses indicated there were a number of “play” responses. For the advantageous decks C and D, there were few “pass” responses. Thus, there were three types of choices: advantageous decks play, disadvantageous decks play, and disadvantageous decks pass. The minimum number of artifact-free trials was in the disadvantageous pass condition (56 trials). We analyzed the modulations by categorizing the trials according to the subsequent types of choice (see Figure 3D). From the figure, we can see that the disadvantageous decks with the response “pass” showed greater P3 amplitude than “play,” particularly in the right hemisphere and the midline.

A 3 (Type of choice: advantageous decks play, disadvantageous decks play, disadvantageous decks pass) × 3 (Region: frontal, central, parietal) × 3 (Laterality: left, middle, right) repeated ANOVA was conducted on P3 amplitude. The results showed that there was a marginally significant main effect of Type of choice, *F*_(2, 50)_ = 3.06, *p* = 0.071, η^2^_*p*_ = 0.109. There was a significant interaction between Type of choice and Region, *F*_(4, 100)_ = 4.19, *p* = 0.021, η^2^_*p*_ = 0.144; and Type of choice and Laterality, *F*_(4, 100)_ = 4.17, *p* = 0.031, η^2^_*p*_ = 0.143; the three-way interaction was also significant, *F*_(8, 200)_ = 3.44, *p* = 0.010, η^2^_*p*_ = 0.121. Simple effect analysis revealed that the main effect of Type of choice was not significant in the left hemisphere, *F*_(2, 50)_ = 1.15, *p* = 0.266, η^2^_*p*_ = 0.058, but was significant in the midline [*F*_(2, 50)_ = 3.60, *p* = 0.049, η^2^_*p*_ = 0.126] and the right hemisphere [*F*_(2, 50)_ = 6.55, *p* = 0.003, η^2^_*p*_ = 0.208]. The disadvantageous decks pass condition had the largest P3 amplitude. At the same time, the interaction between Type of choice and Region showed different patterns across hemispheres; the interaction was significant in the left hemisphere (*p* = 0.004), marginally significant in the midline (*p* = 0.059), and not significant in the right hemisphere (*p* = 0.144). Further analysis showed that the interaction in the left hemisphere indicated that the effect of Type of choice was significant in the parietal region but not in the frontal or central regions; the interaction in the midline indicated that the effect of Type of choice was significant in the central and parietal regions but not in the frontal region. These effects of Type of choice all showed the disadvantageous decks pass condition had larger P3 amplitude than the other two conditions. To summarize, the three-way interaction showed that Type of choice had an effect in specific regions and hemisphere, i.e., in the midline and the right hemisphere; the disadvantageous deck pass showed a larger P3 amplitude.

**Figure 4:**
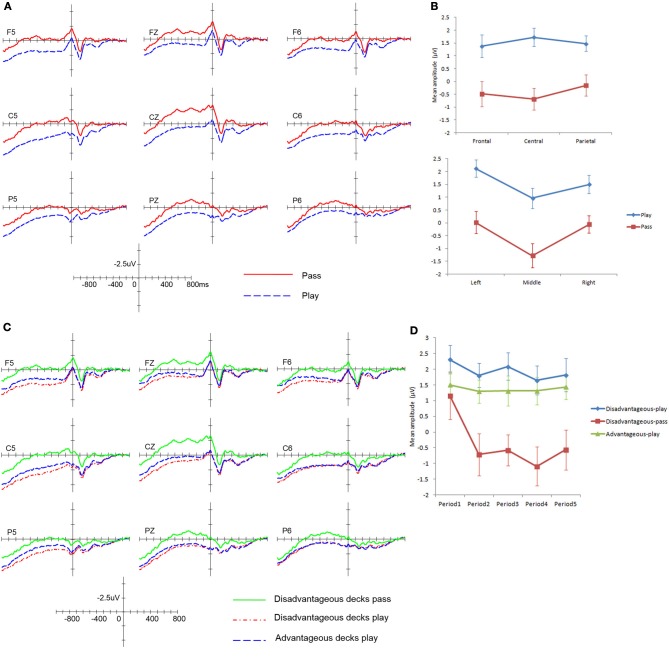
**ERP results in the response selection stage. (A)** ERP modulations with different response types. **(B)** Decision Preceding Negativity amplitudes with different responses. **(C)** ERP modulations with different response types to disadvantageous and advantageous decks. **(D)** Decision Preceding Negativity amplitudes in different periods over the course of the task. Error bars indicate the standard error.

***Response selection stage***. See Figure 4A for the grand average. From the figure, we can see that the response “pass” (press “→”) showed larger negativity, whereas the response “play” (press “←”) showed smaller negativity.

A 2 (Type of choice: play, pass) × 3 (Region: frontal, central, parietal) × 3 (Laterality: left, middle, right) repeated ANOVA was conducted on the mean amplitude of the DPN. The minimum mean number of artifact-free trials among these conditions was 78. The results showed that there was a significant main effect of Type of choice, *F*_(1, 25)_ = 22.60, *p* < 0.001, η^2^_*p*_ = 0.475. There was a significant interaction between Type of choice and Region, *F*_(2, 50)_ = 4.98, *p* = 0.022, η^2^_*p*_ = 0.166; and a significant interaction between Type of choice and Laterality, *F*_(2, 50)_ = 4.00, *p* = 0.036, η^2^_*p*_ = 0.138. The three-way interaction was not significant, *F*_(4, 100)_ = 2.24, *p* = 0.088, η^2^_*p*_ = 0.082.

Simple effect analysis revealed that for the Type of choice × Region interaction, the effect of Type of choice was significant at all frontal, central and parietal regions (*p* < 0.001), with a larger effect at the central region (see Figure 4B left panel). For the Type of choice × Laterality interaction, the effect of Type of choice was also significant in both the left and right hemispheres and the midline (*p* < 0.001); the right hemisphere showed a smaller effect (see Figure 4B right panel). To summarize, the results generally revealed that the “pass” condition showed larger negativity in all regions, and the amplitude was largest in the left and middle regions.

When the type of choice was divided into advantageous decks and disadvantageous decks (see Figure 4C), we found the pattern was consistent with the previous results; the response “pass” for disadvantageous decks showed the largest negativity and was significantly larger than the disadvantageous decks play and the advantageous decks play (*p* < 0.05). Furthermore, similar to the analysis in the choice evaluation stage, we also divided the task into five periods; each period consisted of four blocks (80 trials). A 5 (Period: period 1, period 2, period 3, period 4, period 5) × 3 (Type of choice: disadvantageous deck play, disadvantageous deck pass, advantageous deck play) × 3 (Region: frontal, central, parietal) × 3 (Laterality: left, midline, right) repeated ANOVA was conducted. The results showed that the main effect of Period was significant (*F*_(4, 96)_ = 3.59, *p* = 0.024), η^2^_*p*_ = 0.130. Further analysis showed that the negativity in period 1 was smaller than in periods 2, 4, and 5 (*p* < 0.05). The Period × Type of choice interaction was not significant, *F*_(8, 192)_ = 1.47, *p* = 0.213, η^2^_*p*_ = 0.058. However, there was a trend indicating that the negativity of the disadvantageous deck pass increased largely from period 1 to period 2 and remained relatively stable thereafter, while the disadvantageous play and the advantageous play did not show such a trend (see Figure 4D).

**Figure 5:**
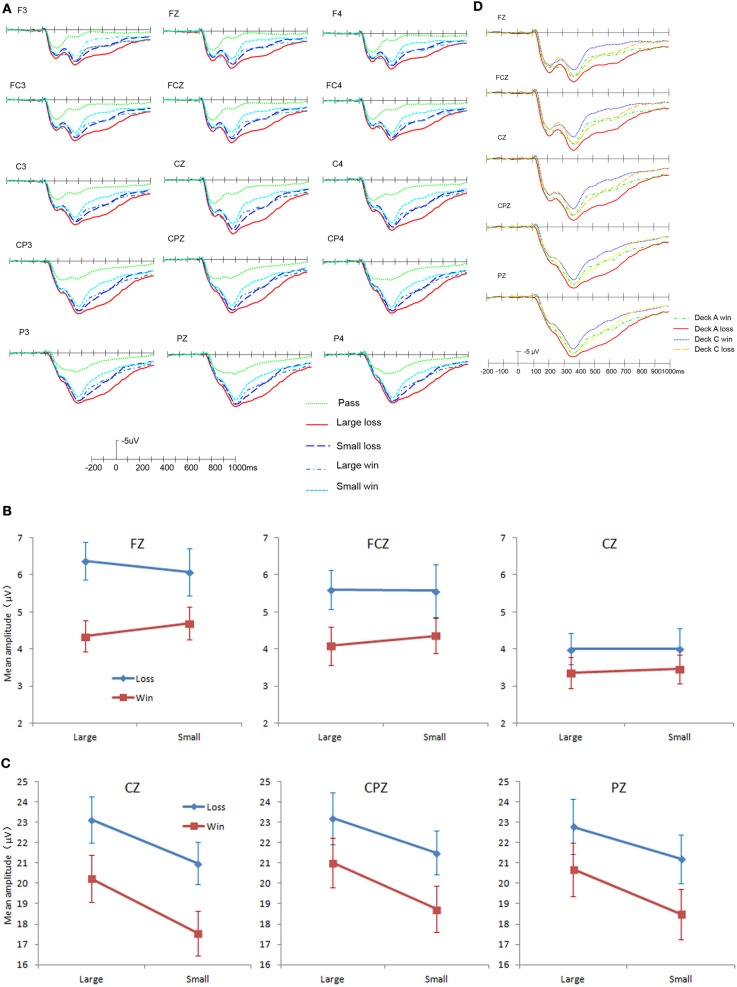
**ERP results in the feedback evaluation stage. (A)** Grand average ERP for different valences and magnitudes in feedback. **(B)** The effects of valence and magnitude on FRN amplitude. **(C)** The effects of valence and magnitude on P3 amplitude. **(D)** Grand average ERP of decks A and C. Error bars indicate the standard error.

***Feedback evaluation stage***. The grand average of the feedback evaluation is presented in Figure 5A. The feedback evoked prominent FRN and P3. The FRN was mainly distributed in the frontal and the central regions, whereas P3 was mainly distributed in the central and the parietal regions. Analyses were conducted to examine the effects of the valence and magnitude of the outcome on the amplitude of these ERP modulations. Main effects of valence, magnitude or interactions involving these variables are presented below.

(1) FRN

A 2 (Valence: win, loss) × 2 (Magnitude: large, small) × 3 (Electrode: FZ, FCZ, CZ) repeated ANOVA was conducted on the FRN amplitude. The minimum mean number of artifact-free trials among these conditions was 80. The results showed that, consistent with prior studies, FRN was sensitive to Valence but not Magnitude. The main effect of Valence was significant, *F*_(1, 25)_ = 7.49, *p* = 0.011, η^2^_*p*_ = 0.230; loss evoked a larger FRN (see Figure 5B). The main effect of Magnitude was not significant, *F*_(1, 25)_ = 0.055, *p* = 0.816, η^2^_*p*_ = 0.002. There was a significant interaction between Valence and Electrode, *F*_(2, 50)_ = 8.77, *p* = 0.001, η^2^_*p*_ = 0.260; the interaction indicated that the difference between a win and loss was largest in the FZ (*p* = 0.002), then in the FCZ (*p* = 0.015), and smallest in the CZ (*p* = 0.150). Other interactions were non-significant.

(2) P3

A 2 (Valence: win, loss) × 2 (Magnitude: large, small) × 3 (Electrode: CZ, CPZ, PZ) repeated ANOVA was conducted on the P3 amplitude. The results showed that there was a significant main effect of Valence, *F*_(1, 25)_ = 31.16, *p* < 0.001, η^2^_*p*_ = 0.555; loss evoked a larger P3. There was a significant main effect of Magnitude, *F*_(1, 25)_ = 14.08, *p* = 0.001, η^2^_*p*_ = 0.360; the larger magnitude evoked a larger P3. There was a significant interaction between Valence and Electrode, *F*_(2, 50)_ = 6.66, *p* = 0.011, η^2^_*p*_ = 0.210, and between Magnitude and Electrode, *F*_(2,50)_ = 6.65, *p* = 0.012, η^2^_*p*_ = 0.210. Other interactions were non-significant. As shown in Figure 5C, the interactions between Valence and Electrode and between Magnitude and Electrode did not affect the main effects.

Considering that the global feedback results might be confounded by the probability of wins and losses, we conducted further analyses by comparing the decks with the same probability of wins and losses. Because decks B and D had few trials of loss and did not have enough trials for averaging, we compared decks A and C, which had 50% wins and losses (see Figure 5D). In Figure 5D, we can see that the ERP modulations showed a similar pattern as the global results. Further analysis also showed that the amplitude of P3 was sensitive to both Valence and Magnitude, while the amplitude of the FRN was only sensitive to Valence.

(3) Correlation analysis between the FRN effect and the P3 amplitude difference between disadvantageous and advantageous decks in the choice evaluation stage

FRN is an ERP modulation specifically related to decision making; it reflects the quick evaluation of whether the results were consistent with the expectation (Wu and Zhou, 2009). Thus, the amplitude difference between wins and losses reflect an individual's ability to discriminate information with different valences in the environment. The evaluation of choice was based on learning from experience, i.e., feedback for choice. Thus, we further analyzed the relationship between the FRN effect (amplitude difference between loss and win) and the amplitude difference between disadvantageous decks and advantageous decks in the choice evaluation stage. The FRN effect was largest in FZ, and the P3 amplitude difference between disadvantageous decks and advantageous decks was also largest in the frontal region. Thus, we calculated the relationship between the FRN effect at FZ and the P3 amplitude difference between the disadvantageous and advantageous decks in the choice evaluation stage at electrode F5 (left hemisphere) and F6 (right hemisphere). The results showed that the FRN effect was negatively correlated with the P3 amplitude difference at F5, *r* = −0.515, *p* = 0.007; the FRN effect was positively correlated with the P3 amplitude difference at F6, *r* = 0.459, *p* = 0.018. These results indicated that the larger the FRN effect, the better the individual discriminated disadvantageous and advantageous decks in the choice evaluation stage.

## Discussion

In the current study, we successfully adopted a revised version of the IGT and used the ERP technique to examine the neural correlates of different stages of uncertain decision making. The main results were as follows: first, in the choice evaluation stage, the advantageous decks and disadvantageous decks were associated with positive and negative somatic representations and showed differential modulations in the brain. Specifically, the advantageous decks evoked a larger P3 in the left frontal hemisphere than the disadvantageous decks, and the disadvantageous decks evoked a marginally significant larger P3 in the right frontal hemisphere than the advantageous decks, consistent with our predictions. These effects extended to the central regions. Second, in the response selection stage, there was a dissociation for the pre-response ERP modulation (DPN) between the responses of “play” and “pass.” The responses of “pass” evoked a larger DPN than “play,” and this effect was mainly caused by the disadvantageous decks. This observation may reflect the fact that the somatic states guided participants to make the response “pass” and is consistent with our predictions. However, the effects extended to the whole brain. Third, in the feedback evaluation stage, FRN was only sensitive to the valence of the outcome, whereas P3 was sensitive to both the valence and the magnitude of the outcome, consistent with our predictions and previous studies.

### Choice evaluation stage

One of the strengths of this study was that we studied the choice evaluation stage of uncertain decision making. Bechara et al. (1996) suggested that the advantageous and disadvantageous decks represented emotional information, such as good or bad, by somatic signaling. However, the temporal resolution of SCRs is low; thus, the nature of the anticipatory SCR is not clear. The two ERP studies (Carlson and Zayas, 2009; Bianchin and Angrilli, 2011) did not clearly examine the choice evaluation stage because they used an IGT version with free choice. In the present study, we adopted a single choice version of the IGT that allowed us to examine the choice evaluation stage.

Results showed that the advantageous decks evoked a greater P3 in the left hemisphere (the frontal and central regions), whereas the disadvantageous decks evoked a greater P3 in the right hemisphere (the central and parietal regions, marginally greater in the frontal region). These results indicated that the advantageous decks were associated with positive somatic representations and the disadvantageous decks were associated with negative somatic representations in the brain. These findings were consistent with the notion of emotive functions with asymmetric cortical activity (Cunningham et al., 2005; Ohgami et al., 2006). Cunningham et al. (2005) asked the participants to make good vs. bad (evaluative) judgments on socially relevant concepts and recorded their late positive potential (LPP) at the same time. The results revealed that the amplitude of the LPP was higher in the left frontal hemisphere when the concepts were rated good and higher in the right frontal hemisphere when the concepts were rated bad. Ohgami et al. (2006) also found that the left hemisphere showed greater activation with monetary rewards. Kayser et al. (1997) found that negative stimuli evoked greater N2 and P3 in the right hemisphere. Graham and Cabeza (2001) found larger frontal modulations when viewing happy faces. The asymmetry effects extended to the central region and partly to the parietal region; this result might be caused by the difference in the nature of the stimuli and the paradigms. Further studies are needed to explore this issue. For example, the cited studies all used explicit emotional stimuli, such as facial expressions, words, and emotional pictures. However, the stimuli in our study consisted of cards without any inherent emotional information; only upon playing with these cards were they associated with positive or negative somatic representations.

Regarding the ERP modulations over the course of the task, the P3 amplitude was not significantly different among the five periods, although we could see the trend of the amplitude (the difference wave between the disadvantageous decks and the advantageous decks) decreasing in the left hemisphere and increasing in the right hemisphere. Although this result was unexpected, it was not unreasonable because it has been found that the rating of “goodness” and “badness” of the decks was above the chance level as early as after the first 20 trials (Bowman et al., 2005; Dunn et al., 2006). In the first period of the present study, participants might have had some sense of the long-term outcomes of the decks and might have associated the decks with somatic representations; thus, participants did not show significant differences for ERP amplitudes in these five periods.

We also analyzed the ERP waveform difference based on the participants' subsequent decisions. The results indicated that for the disadvantageous decks, different decisions had already manifested in the choice evaluation stage. The differences were mainly at the midline and in the right hemisphere; those cards with a subsequent decision of “pass” evoked a larger P3 than those with a decision of “play.” This observation also indicated that the negative somatic representations evoked in the choice evaluation stage would affect the following decisions; the stronger the negative somatic representations, the more likely the participants decided to “pass.”

### Response selection stage

Prior SCR studies showed that the participants presented increased anticipatory SCRs before they made disadvantageous choices; this finding provided important evidence for the theory that emotional signals guided decision making (Bechara et al., 1997). However, it was controversial whether the anticipatory SCRs reflected the anticipation before response selection or before feedback due to the low temporal resolution of this approach. The present study used a technique with high temporal resolution, ERP, and explored the anticipatory effects before the response. The results showed that the response of “pass” evoked a greater DPN than the response of “play.” The global results and the results of the disadvantageous decks all showed the same pattern, and this pattern presented over the whole brain. This modulation reflected the anticipation effect of decision making. Contrary to our hypothesis, the DPN effect was not limited to the right frontal region but spread over the whole brain. It may be that the anticipatory effect was strong and extended to the central and the parietal areas, however, further studies are needed to explore this issue.

The anticipation effect in the present study was somewhat different from the previous SCR studies (Bechara et al., 1996, 1997) and two prior ERP studies (Carlson and Zayas, 2009; Bianchin and Angrilli, 2011); these studies used the free choice version of the IGT. Their results showed that the anticipatory SCR or ERP was greater before participants selected the disadvantageous decks, however, the confounding effect of attention shifting and searching could not be excluded in those studies (Dunn et al., 2006). The results in the present study indicated that the greater DPN was associated with the response “pass”; this finding may indicate that when the designated card evoked a stronger physiological response, the participants felt uneasy and tended to respond “pass.” The results also showed that when participants responded “play,” the advantageous decks and the disadvantageous decks evoked similar DPNs, indicating that if a card did not evoke a significant physiological response, the participants tended to play with the card. This outcome is consistent with the suggestion that frontal cortex activation was associated with identifying negative outcomes and mediates behavioral shifting from disadvantageous choices (Asp et al., 2013). It should be noted that not all disadvantageous decks evoked a larger DPN; this situation may occur because the disadvantageous decks also had some winning cards, although they were disadvantageous decks in the long run. Thus, if the participants perceived the current trial to be safe and did not evoke a physiological response, they would “play”; in contrast, if they perceived the current trial to be risky and evoked a physiological response, they would “pass.” Along the course of the task, they were more aware of the risky decks and selected to “pass” the cards from disadvantageous decks. These results were also consistent with the literature that emotional stimuli modulate readiness for action in a dynamic way; readiness potential was strongly augmented in the emotional condition (Grecucci et al., 2009; van Loon et al., 2010). The results, therefore, support the somatic marker hypothesis (Damasio, 1994) and indicate that there was an anticipatory process that occurred before the response. The present results provided a cortical index of somatic representations on decision making. However, the difference between the SCR and the DPN should be noted. The SCRs were larger for the “play” to disadvantageous decks because selecting from the disadvantageous decks was recognized as a risky choice, in general, after the initial learning phase. The participants could only record the “play” to the disadvantageous decks, whereas merely attending to the disadvantageous decks (finally “play” to the advantageous decks) could not be recorded. For the present DPN results, the DPN was larger for “pass” in the disadvantageous decks after the initial learning phase (from period 2). This effect may occur because if the participants perceived risk from this trial and showed a larger DPN, they would choose to “pass” this card. Thus, the SCR and DPN have some differences, however, they are both indicators of anticipation and have relationships with the subsequent behavioral responses.

### Feedback processing stage

For the feedback evaluation stage, the results of FRN and P3 were consistent with the literature. The P3 was sensitive to both the valence and the magnitude of the outcome (Toyomaki and Murohashi, 2005; Wu and Zhou, 2009). Loss and larger magnitude evoked a greater P3; this outcome was also consistent with Carlson and Zayas (2009), who studied children's decision making with a children's version of the IGT.

The present results indicated that the FRN was only sensitive to valence, which is consistent with previous studies (Gehring and Willoughby, 2002; Yeung and Sanfey, 2004; Toyomaki and Murohashi, 2005; Hajcak et al., 2006). This observation indicated that the FRN reflected the early appraisal of goodness of the results (Yeung and Sanfey, 2004). The results that the FRN amplitude in FZ was negatively correlated with the choice evaluation stage P3 amplitude difference in F5 and positively correlated with that in F6 suggested that the evaluation of choice was related to decision experience from feedback processing. Thus, it was a learning process. One study found that the brain's response to feedback (the FRN) predicted whether subjects learned to avoid an erroneous response the next time, suggesting that FRN amplitudes may reflect motor learning (van der Helden et al., 2010). Our study showed that FRN may also reflect a decision-making learning process.

### Limitations and future directions

There are several limitations of the study. First, we did not record SCRs at the same time. One study recorded magnetoencephalogram (MEG) and SCRs at the same time while participants were presented with emotional and neutral pictures; greater activity was found at 180 ms for the emotional pictures than the neutral ones for the event-related magnetic fields (ERFs) data. There was also a strong association of the early ERF effects and the later SCR effect with emotional aroused stimuli (D'Hondt et al., 2010). If we could record ERP and SCRs at the same time, the relationship between anticipatory SCRs and anticipatory ERP effects could be examined more directly. Second, individual differences may have had some effect on the results (Carlson and Zayas, 2009). However, due to the small sample size of the present study, we could not examine this issue. Future studies should recruit a larger sample size. Third, Damasio (Damasio, 1994) suggested that both emotion and cognition were involved in decision making; one of the functions of somatic markers was to serve as an indicator of the value of response options and as a boost signal for continued working memory and attention processing (Damasio, 1996). However, the present study did not examine the role of cognitive factors in decision making. Thus, further studies are needed to examine this question.

## Conclusions

To conclude, the neural correlates of different stages of uncertain decision making were examined in this study. The results indicated that in the choice evaluation stage, the advantageous decks and the disadvantageous decks were associated with positive and negative somatic representations in the brain. In the response selection stage, participants showed an anticipatory effect. Finally, in the feedback evaluation stage, FRN was only sensitive to the valence of the outcome, while P3 was sensitive to both the valence and the magnitude of the outcome.

## Role of funding sources

This study was supported by grants from the Young Investigator Scientific Fund of the Institute of Psychology, Chinese Academy of Sciences (O9CX073007), the National Key Technologies R&D Program (2012BAI36B01), the National Science Foundation of China (30900403, 81088001 91132701, and 31271106), Youth Innovation Promotion Association Funding of Chinese Academy of Sciences (Y1CX131003), Key Laboratory of Mental Health, Institute of Psychology, the CAS/SAFEA International Partnership Programme for Creative Research Teams (Y2CX131003), the major projects of the Ministry of Education SOCIAL base (12JJD190001), the Beijing Social Science Foundation (12JYA003), and Endeavour Fellowship of Australian government. The funding agents had no further role in the study design, in the collection, analysis and interpretation of the data, in the writing of the manuscript, or in the decision to submit the paper for publication.

### Conflict of interest statement

The authors declare that the research was conducted in the absence of any commercial or financial relationships that could be construed as a potential conflict of interest.

## Footnotes

^1^It should be noted that some researchers thought the IGT should be an ambiguous decision-making task because the participants know the probabilities and magnitudes of the outcomes in a typical uncertain decision making task, whereas in the IGT task, the participants do not know any of these factors.

^2^The outcomes of Deck A included 100, 100, 80, 90, 130, −50, −100, −150, −200, and −250. The outcomes of deck B included 100, 100, 100, 100, 80, 80, 90, 120, 130, and −1150. The outcomes of Deck C included 50, 50, 40, 60, 70, −5, −10, −15, −15, and −25. The outcomes of Deck D included 50, 50, 50, 50, 40, 40, 40, 60, 70, and −200. In the original IGT, the magnitude of win was the same across all trials and only the magnitude of loss varied. Thus, participants only needed to be concerned about the loss. This characteristic implies that the task measures the variance of loss but not the global reinforcement (Dunn et al., 2006). If a card includes both wins and losses at the same time, it would be difficult to distinguish the brain activities associated with a win and loss (Weller et al., 2010). Thus, in the present study, we presented the net score to participants, and both wins and losses varied.

^3^We kept these two ideas in mind when designing the payoff schedules: first, the probability of loss will be 10% for two decks and 50% for the other two decks; second, the magnitude of win will be 100 for two decks and approximately 50 for the other two decks. Thus, it would be difficult to make the expected value of Deck C be 250. In our task, the expected value of Deck C was set to 200.
